# Extraordinary Announcement for 1886

**Published:** 1886-02

**Authors:** 


EXTRAORDINARY ANNOUNCEMENT FOR 1886.—"*You pay your money and take your choice*" of any of the following sterling journals at commutation rates with ours:

DANIEL'S TEXAS MEDICAL JOURNAL, $2, clubbed with *Gaillard's Medical Journal*, $5, the two journals for $5.50!

DANIEL'S TEXAS MEDICAL JOURNAL, $2, clubbed with the *Philadelphia Medical Bulletin*, $1, both for $2.50.

DANIEL'S TEXAS MEDICAL JOURNAL, $2, clubbed with *Atlanta Medical and Surgical Journal*, $2.50, both for $3.50.

DANIEL'S TEXAS MEDICAL JOURNAL, $2, clubbed with *Kansas City Medical Index*, $2, both for $3.

The five leading journals of America, representing the North, East, South and West, all for $9.75.

DR. GAILLARD'S OLD STUDENTS, of whom there are a great many now practicing in Texas, should take advantage of the above reduced rates, and subscribe for the *Journal*, and thus testify their appreciation of their old master and friend. He has five handsome sons coming on to perpetuate his name.

